# The British Hospitals Association in Glasgow

**Published:** 1911-11-18

**Authors:** 


### The British Hospitals Association in Glasgow.

IMPORTANT MEETING ON THE NATIONAL INSURANCE BILL.

A representative meeting of hospital managers and administrators was held in Glasgow on Friday, 10th inst., to consider what further steps should be taken to secure that adequate provision for institutional treatment be made in the National Insurance Bill. Mr. J. D. Hedderwick, D.L., Chairman of the Glasgow Royal Infirmary, who presided, explained briefly the work of the British Hospitals Association to date, so far as the National Insurance Bill is concerned, and said that he understood the medical profession as well as hospital managers were now agreed that if the Insurance Bill was to be workable provision must be made for institutional treatment. The following resolution was moved by Professor Harvey Littlejohn, Edinburgh, seconded by Colonel Roxburgh, Glasgow, and supported by Mr. T. H. Smith, Dundee Royal Infirmary, and Sir William Arrol, Glasgow :—

"That the British Hospitals Association again affirms its unanimous opinion that the National Insurance Bill is incomplete unless it provides for the hospital treatment which insured persons must have if their medical needs are to be covered by the Bill,"

and carried unanimously.

A further resolution was proposed by Mr. W. G. Black, Glasgow Royal and Eye Infirmaries, duly seconded, and carried unanimously :—

"That this meeting records its conviction that hospital treatment as at present provided cannot be adequately maintained by the Scottish hospitals if the existing sources of income from the subscriptions of workmen and employers are seriously diminished, as in the opinion of this meeting they are likely to be in consequence of the provisions of the Bill."

The following hospital representatives were present at the meeting: Professor Harvey Littlejohn, Royal Infirmary, Edinburgh; Lady Susan Gilmour, Edinburgh; Sir William Arrol, Royal Infirmary, Glasgow; Dr. Oliphant, Eye Infirmary, Glasgow; Mr. Peter Watson and Mr. Robert Dempster, Ayr County Hospital; Mr. William Morland and Mr. J. D. Hedderwick, Royal Infirmary, Glasgow; Mr. John Dunn, Victoria Hospital, Glasgow; Mr. W. A. Tait, Edinburgh Royal Infirmary; Mr. W. G. Black, Royal Eye Infirmary, Glasgow; Mr. W. Nisbet, for the Secretary of Royal Infirmary; Mr. R. F. Barclay, Royal Hospital for S.C., Glasgow; Mr. Wm. Cross, Royal Infirmary, Glasgow; Mr. J. Neilson Gray, Lock Hospital, Glasgow; Mr. R. G. Paterson, Eye Infirmary, Glasgow; Professor John Glaister, Royal Infirmary, Glasgow; Mr. Wm. Gray, Victoria Infirmary, Glasgow; Mr. Jas. A. Love, Secretary, Greenock Infirmary; Mr. J. Macpherson, Western Infirmary, Glasgow; Mr. John Glen, Royal Infirmary, Glasgow; Mr. J. Spenser, Mr. W. Clapperton, and Mr. W. J. Tillie, Eye Infirmary, Glasgow; Mr. J. Birkmeyse, jun., Broxstone Jubilee, Port Glasgow; Mr. Wm. Cockran, Eye Infirmary; Mr. J. Armour Brown, Paisley Infirmary; Mr. A. Scott Finnie, Royal Infirmary, Aberdeen; Mr. Jas. H. Young, Paisley Infirmary; Mr. G. Ross, Western Infirmary, Glasgow; Mr. R. Kinlock and Dr. E. L. Paton, Royal Infirmary, Perth; Mr. James Macfarlan, and Mr. J. Maxtone Thom, Royal Infirmary, Glasgow; Mr. N. Service, Eye Infirmary; Mr. Jas. A. D. Mackean, Paisley Infirmary; Mr. John Edwards, Eye Infirmary, Glasgow; Mr. J. A. Roxburgh, Western Infirmary; Mr. James S. Craig, Royal Infirmary, Glasgow; Mr. Arthur Hail, Mr. James Macara, and Dr. Bruce Goff, Western Infirmary; Mr. T. H. Smith (Chairman), and Dr. H. C. Fraser, Dundee Royal Infirmary; Mr. T. Bisset, Dr. John Stewart, Dr. Macgregor, and Mr. W. Bruce, Victoria Infirmary, Glasgow; Mr. Arthur Forbes (Secretary), Maternity Hospital; Mr. Henry Leash, Victoria Infirmary, Glasgow.

---

THE INSURANCE BILL AND CARDIFF INFIRMARY.

The resolution passed at the conference of the British Hospitals Association, asking for the Chancellor of the Exchequer's consideration of the interests of the hospitals in relation to the National Insurance Bill, was unanimously adopted at their monthly meeting by the Board of Management of King Edward VII.'s Hospital, Cardiff (the Cardiff Infirmary). Copies of the resolution, as so adopted, were sent to thirty members of Parliament, either representing constituencies served by the hospital or themselves personally interested in the locality. The latter include Mr. Donald Maclean, member for Peebles and Selkirk, the newly-appointed Deputy Chairman of Committees, and Mr. Harry Webb, the member for the Forest of Dean. Mr. Maclean's brother, Dr. Ewen Maclean, is the senior Gynæcologist of the hospital, and Mr. Harry Webb, who has a residence within a few miles of Cardiff, is a generous benefactor of the institution. The modern casualty department, as well as the first operating-theatre, was the gift of Mr. Webb's uncle, the late Mr. Thomas Webb. It is a matter for sincere regret that the member for Cardiff, Lord Ninian Crichton Stuart, is at present seriously ill. Lord and Lady Ninian have taken a personal interest in the Cardiff Hospital, and have recently endowed a bed in the children's ward. This policy of making known the resolution is one that was strongly recommended at Manchester.

